# Crohn’s disease manifesting as ileo-urachal fistula: Two cases reports and review of literatures

**DOI:** 10.1016/j.ijscr.2018.10.031

**Published:** 2018-10-26

**Authors:** Hirosuke Kuroki, Akira Sugita, Kazutaka Koganei, Kenji Tatsumi, Ryo Futatsuki, Nao Obara, Katsuhiko Arai, Tsuneo Fukushima

**Affiliations:** a Department of Surgery, Yokohama Municipal Citizen’s Hospital, 240-8555 56, Okazawacho Hodogaya-ku, Yokohama City, Japan; b Matsushima Clinic, 220-0045 3-138 Isecho Nishi-ku, Yokohama City, Japan

**Keywords:** Crohn’s disease, Urachus, fistula

## Abstract

Introduction: A fistula involving a patent urachus in a patient with Crohn’s disease is rare. Here we report ileourachal fistula formation in two patients with Crohn’ disease.

Case presentations: The first patient was a 29-year-old man with Crohn’s disease and ileitis, and the second patient was a 43-year-old man with Crohn’s disease and ileitis. One of the patients showed pus/fecal discharge via the umbilicus. Both patients were eventually diagnosed with an ileourachal fistula associated with Crohn’s disease. In the first patient, the urachal remnant was connected to the urinary bladder and a Crohn’s disease-related intestinal lesion had formed a fistula to the urachus. In the second patient, a periumbilical inflammatory lesion extended to the bladder through the urachal remnant and to a longitudinal ulcer of the ileal lesion. The first patients underwent partial ileal resection, and partial cystectomy, while the second patient underwent urachal curettage, partial ileal resection, and partial cystectomy. In both.

Conclusion: In cases of Crohn’s disease with an enterocutaneous fistula or pus discharge via the umbilicus, an examination to detect an urachal remnant with a fistula from the diseased intestine should be performed.

## Highlights

• Urachal tumor or umbilical discharge in Crohn’s disease are examined for internal fistula from diseased ileum to urachus.
• En broc resection with intestinal lesion and urachus is performed with successful outcome.
• Partial cystectomy is sometimes performed with urinary bladder inflamed.

## 1 Introduction

Crohn’s disease usually manifests as urinary complications, including fistulas. When a fistula develops near the umbilicus, a tract or inflammation can potentially form remnant embryonic structures, including a patent urachus. There are few reports in the global medical literature on a fistula involving a patent urachus in a patient with Crohn’s disease [1]. Here, we report ileourachal fistula formation in two patients with Crohn’s disease and describe the clinical features, treatments,and outcomes of these partients. The finding have been reported in line with the PROCESS criteria [2].

## 2 Presentation of cases

### 2.1 Case 1

**Fig. 1:**
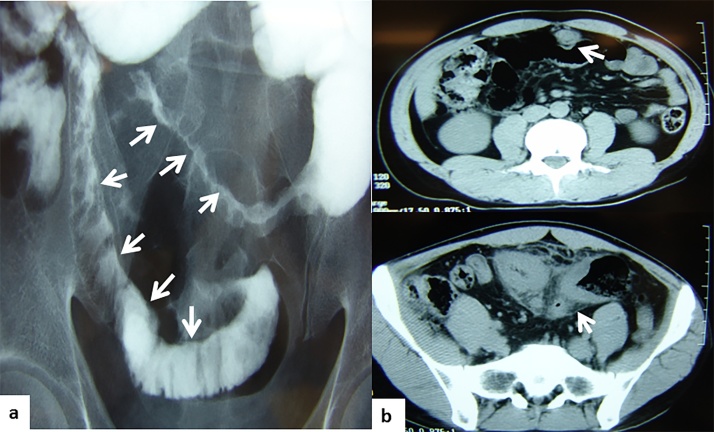
a) A longitudinal ulcer is noted in a small bowel series. b) urachal mass extends into the umbilicus on computed tomography.

**Fig. 2:**
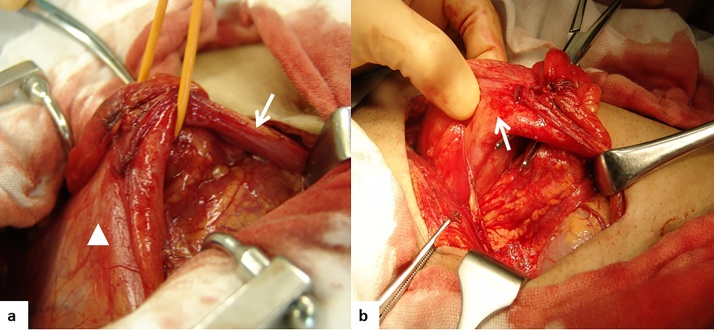
a) A remnant urachus and the urinary bladder are seen. An intestinal lesion adheres to the urachus around the urinary bladder, and the remnant urachus appears swollen. b) The inflammatory mass includes the urachus and intestinal lesion.

**Fig. 3:**
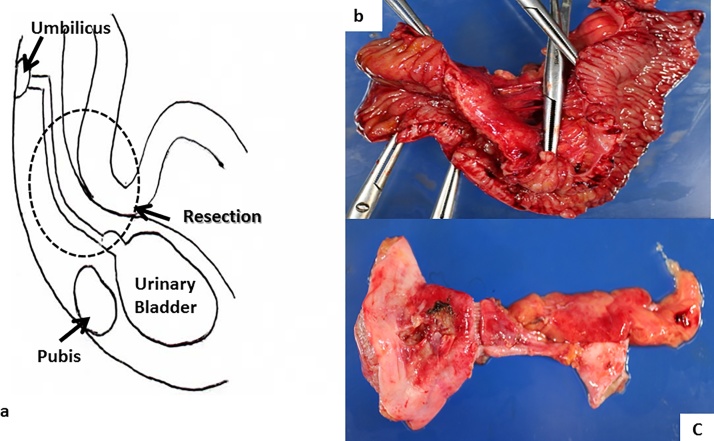
Operative procedure and resected specimen. a) Operative findings. b) Ileum with Crohn’s disease. c) Abscess formation in the urachus. The extent of resection of the shema is part of the circle. Partial ileal resection, urachal resection, and partial cystectomy were performed. Reconstruction involved ileal end to end anastomosis.

**Fig. 4:**
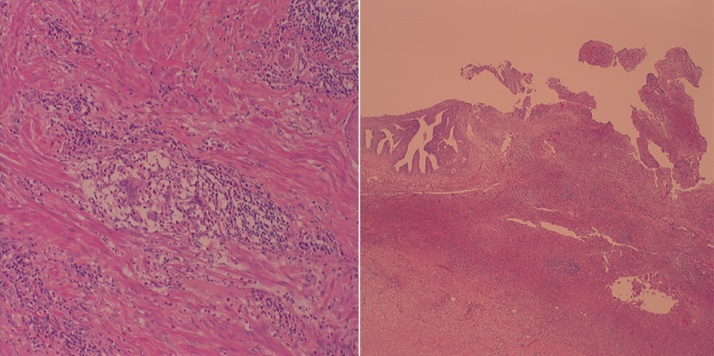
a) Non-caseating epithelioid granulomas in the muscularis propria of the small intestine and inflammation of all organ layers are seen. These findings are which is a characteristic of Crohn’s disease. b) Extensive neutrophil inflammation and a strong inflammatory response to foreign substances beneath the epithelium of the urachus are seen.

A 29-year-old man with Crohn’s disease and ileitis experienced upper abdominal pain. A small bowel series identified a longitudinal ulcer and subsequent abdominal computed tomography (CT) showed an ileal stricture and an urachal tumor mass that extended into the umbilicus (Fig. 1a, b). He was referred to our hospital for surgery because of a fistula from the stenotic ileum to the urachal tumor. Intraoperatively, it was found that the urachal remnant connected to the urinary bladder (Fig. 2a, b), and the Crohn’s disease-related intestinal lesion had formed a fistula to the urachus. The patieint underwent a partial ileal resection, urachal resection, and partial cystectomy (Fig. 3a–c). There were non-caseating epithelioid granulomas in the muscularis propria of the small intestine with inflammation of all layers, and these findings are characteristic of Crohn’s disease. There was extensive neutrophil infiltration and inflammation beneath the epithelium of the urachus because of exposure to foreign substances (Fig. 4a, b). The patient had an uneventful recovery and was healthy with no recurrence at 8 years of follow-up.

### 2.2 Case 2

**Fig. 5:**
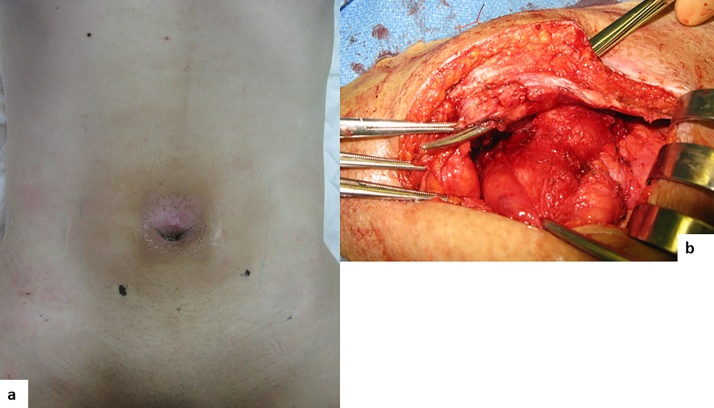
a) Inflammation of the umbilicus. b) Operative findings show a periumbilical inflammatory lesion extending to the bladder through the urachal remnant and to the ileal lesion.

A 43-year-old man was diagnosed with Crohn’s disease and ileitis when he was at 33 years of age. At 41 years of age, he experienced fecal discharge from his umbilicus, pneumaturia, and fecaluria with frequent urination. A small bowel series and barium enema study showed an ileal stricture, and an ileorectal fistula. The patient was referred to our institute for surgery at 43 years of age. A small bowel series showed an ileal lesion with a fistula to the rectum and cecum, but not to the umbilicus. Abdominal CT indicated an ileal lesion involving the urachus, with abscess formation. Because the patient had severe pain, cystoscopy could not be performed. Intraoperatively, it was found that the patient had a periumbilical inflammatory lesion extending to the bladder through the urachal remnant and the longitudinal ulcer of the ileal lesion (Fig. 5a, b). The patient underwent urachal curettage, partial ileal resection, and partial cystectomy. The patient showed severe inflammation of the bladder, a decreased bladder capacity of 150 ml after the cystectomy, and frequent urination. He was administered infliximab after the surgery and was free of recurrence at 6 years of follow-up.

## 3 Discussion

The urachus is derived from the endoderm, and it extends upward from the top of the fetal bladder. The urachal lumen typically closes after approximately 5 months of gestation. The incidence of incomplete obliteration to has been reported be 1 in 5000, and it leads to an anatomical abnormality [3]. Internal fistulization is a common complication of Crohn’s disease. Internal fistulas form between adjacent bowel loops or between the bowel and bladder or vagina [4]. These fistulous tracts occur secondary to transmural inflammation and abscess formation. Among all the fistulas to the urinary system in Crohn’s disease, enterovesical fistulas represent 8–12% of internal fistulas, while enterourachocutaneous fistulas represent 0.6–1% only of these fistulas [4]. In Crohn’s disease, enterourachal fistula formation involves the spread of the fistula to the urachus through the bladder [5] and subsequent spread of spontaneous umbilical fistulization along the course of a persistent urachal remnant [1,4,5].

Patients with an ileourachal fistula have a history of pneumaturia, fecaluria, umbilical drainage from the umbilicus, and cystitis symptoms [5]. The diagnostic work-up in these patients should include ultrasound, CT, MRI, cystoscopy, a small bowel series, fistulography, or a barium enema study [4,5]. In particular, CT reveals the form of the soft tissue mass like cyst from the umbilicus to the apex of the bladder, and recognition of the effect of the wall with enhancement [3]. There are few reported cases of an enterourachal fistula diagnosed with a small bowel series, fistulography, and a barium enema study [1,5]. Many authors have reported that the initial diagnosis was confirmed during exploratory surgery [3,[5], [6], [7]]. Additionally, it has been mentioned that nutritional support or medical therapy is important in the treatment of an enterourachocutaneous fistula [7].

In many of these cases, urachal resection and partial cystectomy with intestinal resection are performed. Urachal resection is generally recommended because there is a 30% chance of urachus reinfection and because the urachal sinus has malignant potential [8]. Several authors have suggested that primary urachal adenocarcinoma may result from either embryonic metaplasia of the transitional cell lining or inclusion of the colonic mucosa [3]. Surgical excision of an enterourachal fistula is considered as one treatment options.

Seventeen cases have been reported globally, including our cases (Table 1) [1,[3], [4], [5], [6], [7], [8], [9], [10], [11]]. There was only three reported cases only of pyourachus in patients with Crohn’s disease in Japan [11], including both of our cases. Fourteen cases had an ileal lesion. Ileal resection was performed in all cases, except one of our cases. One urachal cyst was ablated, and one case was treated with medical therapy alone. There was no experienced recurrence of the urachal lesion.

**Table 1:** Case reports for urachal lesion of Crohn's disease.

Author	Age	Sex	Syptoms related to umbilicus	Preoperative diagnosis related to urachus	Culprit intestine	Treatment for urinary systems
1. Davidson	28	M	feces from umbilicus	urachal abscess	ileum	urachal resection partial cystectomy
2. Goldman	3	M	supra pubic pain dysuria	ruptured appendix	appendix	urachal resection partial cystectomy
3. Solem	n/d	n/d	n/d	urachal abscess	ileum	n/d
4. Solem	n/d	n/d	n/d	urachal abscess	ileum	n/d
5. Klineberg	19	M	umbilical discharge	urachal abscess	ileum	urachal resection partial cystectomy
6. Artigas	20	F	umbilical discharge	urachal fistula	ileum	urachal resection
7. Velosa	19	F	umbilical discharge	enterourachal fistula	transverse colon	medicine
8. Velosa	18	F	umbilical discharge	enterourachal fistula	ileum	medicine
9. Hiley	30	M	umbilical discharge	enterourachal fistula	ileum	fecal diversion
10. Hiley	23	F	umbilical discharge	enterourachal fistula	transverse colon	fecal diversion
11. Hiley	16	F	feces from umbilicus	enterourachal fistula	ileum	medicine(PSL)
12. Hiley	13	F	umbilical discharge	enterourachal fistula	ileum	intermittent drainage
13. Rentz	19	M	umbilical mass	urachal tumor	ileum	urachal resection partial cystectomy
14. Brett	11	F	umbilical discharge	enterourachal fistula	ileum	urachal resection
15. Tsukui	31	F	abdominal pain	urachal abscess	cecum	urachal resection partial cystectomy
16. Case 1	29	M	abdominal pain	urachal abscess	ileum	urachal resection partial cystectomy
17. Case 2	43	M	umbilical discharge pneumaturia fecaluria	enterourachal fistula	ileum	urachal resection urahal curettage partial cystectomy

n/d : no description.

RLQ : right lower quadrant.

PSL : prednisolone.

## 4 Conclusion

In cases of Crohn’s disease with an enterocutaneous fistula or pus discharge via the umbilicus an examination to detect an urachal remnant with a fistula from the diseased intestine should be performed. En bloc resection of the urachal remnant and intestinal lesion is a viable surgical treatment option if an urachal remnant and intestinal lesion are identified.

## Conflicts of interest

The authors declare no conflicts of interest associated with this manuscript.

## Funding source

None of the authors has any conflicts of interest or any financial ties to disclose.

## Ethical approval

We have a consent by the patient. Ethical approval was obtained from the ethical committee of Hiroshima University Hospital.

## Consent

Written informed consent was obtained from the patients for the publication of this case report and any accompanying images. A copy of the written informed consent is available for review by the Editor-in-Chief of this journal.

## Author contribution

HY and KT wrote the manuscript. KT, RH, YS and HO performed the operation. HY and KT performed the research/study, analyzed the data, designed the study, and interpreted the results. All authors conceived the study, participated in its design and coordination, and helped draft the manuscript. All authors read and approved the final manuscript.

## Registration of research studies

We have registered for Research Registry. (UIN:researchregistry4274).

## Guarantor

Dr Tanbe.

## Provenance and peer review

Not commissioned, externally peer reviewed.
